# Polygenic risk for schizophrenia and measured domains of cognition in individuals with psychosis and controls

**DOI:** 10.1038/s41398-018-0124-8

**Published:** 2018-04-12

**Authors:** Rebecca Shafee, Pranav Nanda, Jaya L. Padmanabhan, Neeraj Tandon, Ney Alliey-Rodriguez, Sreeja Kalapurakkel, Daniel J. Weiner, Raquel E. Gur, Richard S. E. Keefe, Scot K. Hill, Jeffrey R. Bishop, Brett A. Clementz, Carol A. Tamminga, Elliot S. Gershon, Godfrey D. Pearlson, Matcheri S. Keshavan, John A. Sweeney, Steven A. McCarroll, Elise B. Robinson

**Affiliations:** 1 Department of Genetics, Harvard Medical School, Boston, MA USA; 2 Stanley Center for Psychiatric Research, Broad Institute of MIT and Harvard, Cambridge, MA USA; 3 College of Physicians & Surgeons, Columbia University Medical Center, New York, NY USA; 4 Department of Psychiatry and Cognitive Neurology, Beth Israel Deaconess Medical Center, Boston, MA USA; 5 Baylor College of Medicine, Texas Medical Center, Houston, TX USA; 6 Department of Psychiatry and Behavioral Neuroscience, University of Chicago, Chicago, IL USA; 7 Program in Medical and Population Genetics, Broad Institute of MIT and Harvard, Cambridge, MA USA; 8 Analytic and Translational Genetics Unit, Department of Medicine, Massachusetts General Hospital and Harvard Medical School, Boston, MA USA; 9 Harvard Medical School, Boston, MA USA; 10 Department of Psychiatry, University of Pennsylvania, Philadelphia, PA USA; 11 Department of Psychiatry and Behavioral Sciences, Duke University Medical Center, Durham, NC USA; 12 Department of Psychology, Rosalind Franklin University of Medicine and Science, North Chicago, IL USA; 13 Department of Experimental and Clinical Pharmacology, University of Minnesota, Minneapolis, MN USA; 14 Department of Psychiatry, University of Minnesota, Minneapolis, MN USA; 15 Department of Psychology and Neuroscience, University of Georgia, Athens, GA USA; 16 Department of Psychiatry, University of Texas Southwestern Medical Center, Dallas, TX USA; 17 Department of Human Genetics, University of Chicago, Chicago, IL USA; 18 Departments of Psychiatry and Neurobiology, Yale University and Olin Neuropsychiatric Research Center, Hartford, CT USA; 19 Department of Psychiatry, Beth Israel Deaconess Medical Center and Massachusetts Mental Health Center, Harvard Medical School, Boson, MA USA; 20 Department of Psychiatry and Behavioral Neuroscience, University of Cincinnati, Cincinnati, OH USA; 21 Department of Epidemiology, Harvard T.H. Chan School of Public Health, Boston, MA USA

## Abstract

Psychotic disorders including schizophrenia are commonly accompanied by cognitive deficits. Recent studies have reported negative genetic correlations between schizophrenia and indicators of cognitive ability such as general intelligence and processing speed. Here we compare the effect of polygenetic risk for schizophrenia (PRS_SCZ_) on measures that differ in their relationships with psychosis onset: a measure of current cognitive abilities (the Brief Assessment of Cognition in Schizophrenia, BACS) that is greatly reduced in psychotic disorder patients, a measure of premorbid intelligence that is minimally affected by psychosis onset (the Wide-Range Achievement Test, WRAT); and educational attainment (EY), which covaries with both BACS and WRAT. Using genome-wide single nucleotide polymorphism (SNP) data from 314 psychotic and 423 healthy research participants in the Bipolar-Schizophrenia Network for Intermediate Phenotypes (B-SNIP) Consortium, we investigated the association of PRS_SCZ_ with BACS, WRAT, and EY. Among apparently healthy individuals, greater genetic risk for schizophrenia (PRS_SCZ_) was significantly associated with lower BACS scores (*r* = −0.17, *p* = 6.6 × 10^−4^ at P_T_ = 1 × 10^−4^), but not with WRAT or EY. Among individuals with psychosis, PRS_SCZ_ did not associate with variations in any of these three phenotypes. We further investigated the association between PRS_SCZ_ and WRAT in more than 4500 healthy subjects from the Philadelphia Neurodevelopmental Cohort. The association was again null (*p* > 0.3, *N* = 4511), suggesting that different cognitive phenotypes vary in their etiologic relationship with schizophrenia.

## Introduction

Schizophrenia is a debilitating psychiatric disorder that commonly involves severe cognitive deficits that compromise functional ability^1,2^. Underperformance in general intelligence tasks as well as tasks designed to be specific to cognitive domains such as memory, executive function, and motor function have been noted in psychosis patients^3^.

Many schizophrenia-associated cognitive deficits are present many years prior to the onset of the illness^4,5^. A meta-analysis of 4396 schizophrenia cases and 745,000 controls showed that every point decrease in premorbid IQ associated with a 3.7% increase in schizophrenia risk^6^. In a nationwide cohort of over 900,000 Swedish individuals, children with the lowest grades showed a 4-fold increased risk of developing schizophrenia and schizoaffective disorder and a 3-fold increased risk of developing other psychotic illnesses^7^. Additionally, studies of clinically high-risk (CHR) groups have shown that people with attenuated psychotic symptoms were cognitively impaired compared to healthy controls (HC) and that, within the CHR group, those that converted to a chronic psychotic disorder within one or 2 years of ascertainment displayed lower cognitive performance compared to those that did not convert^8–11^. Together these results indicate that cognitive deficits are significantly associated with risk of developing a psychotic illness.

Both cognitive performance and psychotic disorders such as schizophrenia are heritable^12–19^, and significant genetic overlap has been consistently reported between schizophrenia and some indicators of cognitive ability, such as general intelligence or processing speed^20–26^. However, it is still unclear how the genetic differences associated with schizophrenia influence cognitive function, and which domains of cognitive function are most associated with schizophrenia risk.

Motivated by these earlier findings, we investigated the relationship between polygenic risk for schizophrenia—as defined by large constellations of common variants that associate with schizophrenia risk (PRS_SCZ_)—and three cognitive phenotypes in the Bipolar-Schizophrenia Network for Intermediate Phenotypes^27,28^ (B-SNIP) cohort: (1) the Brief Assessment of Cognition in Schizophrenia (BACS)^29^, which provides a composite score of current general cognitive function; (2) the Wide-Range Achievement Test (WRAT)^30–32^ reading score, a measure of premorbid intellectual potential; and (3) educational attainment (as measured by years of education, EY). These phenotypes are correlated but differentially associated with psychosis-spectrum case status. Compared to BACS or general cognition, WRAT scores are minimally affected by psychosis onset^8^, and are commonly used as a measure for premorbid intelligence in people with psychotic disorders^30–32^; educational attainment is phenotypically associated with WRAT and BACS and also strongly genetically overlaps with cognition^33,34^. A companion analysis was conducted in the large Philadelphia Neurodevelopmental Cohort (PNC, *N* = 4511)^35-37^ investigating the relationship between WRAT and PRS_SCZ_ since WRAT measures were also available in the PNC.

As an additional validation analysis we investigated the relationship between the polygenic score of educational attainment (PRS_EDUC_) and these three cognitive phenotypes because of the significant genetic overlap between educational attainment and cognition^25,34^.

## Methods

### Study design and participants

Demographic information about the B-SNIP and the PNC cohorts can be found in Table 1. The B-SNIP analysis included 737 Caucasians from the Bipolar-Schizophrenia Network for Intermediate Phenotypes (B-SNIP)^27,28^, which is a five-site consortium (Maryland Psychiatric Research Center, University of Chicago/University of Illinois at Chicago, University of Texas-Southwestern, Wayne State University/Harvard University, and the Institute of Living/Yale University) organized to address questions about diagnostic boundaries and familiality of intermediate phenotypes. Previous work using this cohort reported BACS performance to be consistent with a dimensional model of psychosis^27,38^; Hill et al. (2013) showed that cognitive performance declined progressively as affective symptoms became less prominent and psychotic features became more pronounced and pervasive. Due to these findings, we combined all psychotic probands to form the PSYCH group (*N* = 314) consisting of schizophrenia (*N* = 100), psychotic bipolar disorder (*N* = 143), and schizoaffective disorder patients (*N* = 71). The NPSYCH group consisted of unrelated nonpsychotic individuals combining samples collected as controls (HC, *N* = 180) and first-degree relatives of probands with no history of psychosis (NPFAM, *N* = 243) and without elevated axis II traits^27^ (cluster A or cluster B). While the NPFAM members of the NPSYCH group were related to probands in the PSYCH group, none of the analyses included related individuals (e.g., group differences were calculated between HC and PSYCH or between NPFAM and HC; correlation analyses with PRS_SCZ_, PRS_EDUC_ or between the three cognitive phenotypes were conducted within the PSYCH and the NPSYCH groups separately). All participants provided written informed consent. Institutional review boards at each site approved the study and all sites used identical diagnostic, clinical, and recruitment techniques^28^.

**Table 1:** Demographic information for the B-SNIP and PNC cohorts

	B-SNIP			PNC
	NPSYCH		PSYCH	Controls
	HC	NPFAM		
*N*	180	243	314	4511
Age (years)	38.7 (12.8)	46.5 (14.5)	34.9 (1.3)	13.8 (3.7)
Sex (%F)	51.6	73.7	45.9	50.0
Years of education	15.2 (2.5)	14.9 (2.5)	13.9 (2.3)	N/A

*B-SNIP* Bipolar-Schizophrenia Network for Intermediate Phenotypes, *PNC* Philadelphia Neurodevelopmental Cohort, *NPSYCH* B-SNIP nonpsychotic group consisting of healthy controls (HC) and nonpsychotic relatives (NPFAM), *PSYCH* B-SNIP psychotic proband group consisting of schizophrenia (*N* = 100), psychotic bipolar (*N* = 143), and schizoaffective disorder (*N* = 71) patients. Mean values are shown with standard deviations in parentheses. Years of Education was not an applicable measure for the young PNC cohort. Only samples with European ancestry were used in this study.

The Philadelphia Neurodevelopmental Cohort (PNC) is a sample from the greater Philadelphia area, including over 9000 individuals aged 8–21 years who received medical care at the Children’s Hospital at Philadelphia network^35–37^. The overall inclusion criteria for the cohort included: (1) Ability to provide signed informed consent (parental consent was required for participants under age 18), (2) English language proficiency, and (3) Physical and cognitive ability to participate in computerized cognitive testing. Only unrelated participants (pi-hat <0.2) of European ancestry were used in this work. Individuals with significant medical conditions that can impact brain function, as well as those with either an invalid or incomplete neurocognitive battery were excluded. After genetic quality control (described below and in Supplementary Material) the final sample for this study consisted of 4511 unrelated individuals (mean age 13.76 years, S.D. 3.66 years). All analyses in the PNC cohort were done in this entire sample.

### Cognitive measures

Three cognitive measures were available in the B-SNIP cohort: BACS, WRAT, and educational attainment. General cognitive function in the B-SNIP was measured by the BACS, which is a 30 min test of global neuropsychological function^29^. Premorbid intellectual potential was measured using the reading score of the Wide-Range Achievement Test (WRAT IV), which has a phenotypic correlation of ~0.4 with full-scale intelligent quotient^30,39^. Self-reported years of education completed at the time of recruitment was used as a measure of EY. WRAT was similarly assessed in the PNC sample. A BACS equivalent was not available in the PNC and due to the young age of the subjects (8–21 years) EY would be largely redundant to age itself.

### Genetic analyses

Genetic data for the B-SNIP project were collected for 2053 subjects (multi-ethnic sample) using the Illumina Infinium PsychArray BeadChip™ platform. Genotypes underwent quality control using PLINK 1.9^40,41^ based on a standardized protocol^42^ (Supplementary Material). After initial quality control, and removal of individuals with missing cognitive phenotypes, 1528 samples remained of whom 927 were self-reported Caucasians (SRC). To avoid population stratification, only SRC samples were used in all analyses. The ancestries of these SRC samples were verified by principal component analysis combining the B-SNIP genotype data with the 1000 Genomes phase 1 data^43^. Samples that were more than four standard deviations away from the SRC group mean along the first ten principal components were excluded resulting in a final sample size of 737 (Figure S1). Imputation of the B-SNIP genetic data was performed using HAPI-UR for pre-phasing^44^ and IMPUTE2 for imputation^45,46^ using a multi-ethnic (the 1000 Genomes phase 1 reference panel^43^) reference panel^47^. Poorly imputed single nucleotide polymorphisms (SNPs) were filtered post-imputation (SNPs with information score <0.5^48^ were removed) resulting in 22.5 million imputed SNPs.

Genotype data for 8211 multi-ethnic PNC samples were downloaded from dbGAP. These data were distributed across five different Illumina genotyping chips (as described in the Supplementary Material). Quality control was performed with the programs PLINK^41^ and GCTA^49^. After principal component analysis of the PNC data combined with the HapMap reference panel^50^, only samples with European ancestry were retained by visual inspection (overlapping with CEU and TSI, Figure S2). Following these steps 4733 samples and 204,597 markers were retained for imputation. The Michigan Imputation Server^51^ was used for genetic imputation of the PNC data (Minimac3^51^ for imputation and HAPI-UR^44^ for phasing) with the 1000 genome phase 3 data^52^ as reference panel resulting in a total of 18 million imputed markers. The imputed variants were filtered for info score ≥0.6 (7.9 million markers) for polygenic score calculation with PLINK. Filtering samples for medical criteria and missing cognitive phenotypes (see Study Design and Participants) resulted in a final PNC sample of 4511 unrelated healthy individuals.

Schizophrenia polygenic profile scores (PRS_SCZ_) and educational attainment polygenic scores (PRS_EDUC_) were calculated using the schizophrenia GWAS summary statistics of the Psychiatric Genome Consortium (PGC)^19^ (https://www.med.unc.edu/pgc/results-and-downloads) and the summary statistics from Okbay et al.^34^, respectively. Score calculation was done using custom scripts in the B-SNIP and using PLINK in the PNC. Of the 120,636 PGC schizophrenia polygenic score training SNPs, 101,927 overlapped with the imputed B-SNIP data and 85,598 overlapped with the imputed PNC data. Of the 626,000 educational attainment GWAS markers (clumped using the 1000 Genome^43^ European Ancestry group; *r*^2^ < 0.1 within a 500 kb window of a more significantly associated SNP), 530,894 and 210,501 SNPs were in common with the imputed data in B-SNIP and the PNC, respectively. Polygenic scores were calculated for seven *p*-value thresholds of significance of association: *P* ≤ 10^−4^, 0.001, 0.01, 0.05, 0.1, 0.5, and 1.0. The first 10 principal components from ancestry analyses of B-SNIP and PNC were used as covariates for correlation analyses in the respective cohorts.

### Statistical analyses

**Fig. 1:**
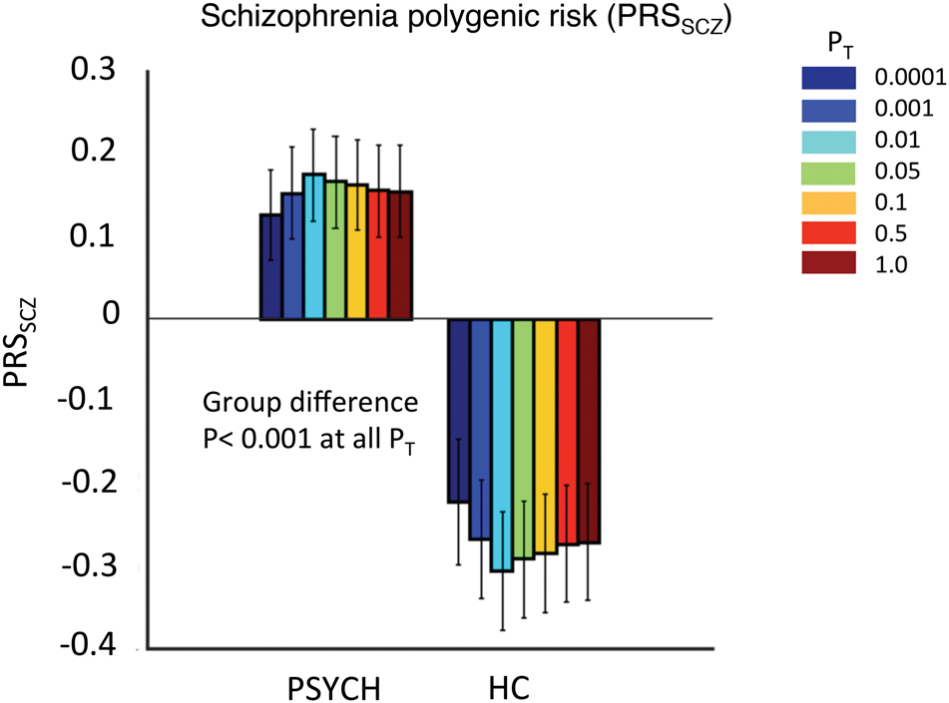
Mean Polygenic scores of schizophrenia (PRS_SCZ_) in the psychotic (PSYCH, *N* = 314) and the healthy controls (HC, *N* = 180) in B-SNIP. The vertical black lines show the standard errors of the mean (SEM). Scores were calculated at seven *p*-value thresholds (P_T_): 0.0001, 0.001, 0.01, 0.05, 0.1, 0.5, and 1.0 (shown in different colors). All scores were z-transformed before mean and SEM calculation. PRS_SCZ_ was significantly higher (*p* ≤ *P*_FDR_ = 2.6 × 10^−^^4^, Kruskal–Wallis test) in the PSYCH group compared to the HC group at all P_T._ Table S1 shows the *p*-values for this analysis. NPFAM (nonpsychotic family members of PSYCH group probands) were excluded from this case-control analysis so that only unrelated individuals were compared

All statistical analyses in B-SNIP were performed using Matlab (version 2012b). Correlations between BACS, WRAT, and EY and the polygenic scores were calculated within the PSYCH group and the NPSYCH group (HC + NPFAM) separately using the Spearman Rank method, which is a nonparametric measure of correlation (deviation from normal distribution was noted in WRAT, EY, and PRS_SCZ_ in specific groups). Age, sex, data collection site, the first 10 principal components from the genetic ancestry analysis, and DSM diagnosis (schizophrenia/bipolar disorder/schizoaffective disorder status for members of the PSYCH group and respective relative’s diagnosis for member’s of the NPFAM group) were regressed out for correlation analyses within each group. As an additional precaution, the samples’ HC/NPFAM status was used as a covariate for all analyses within the NPSYCH group. Differences in BACS, WRAT, and EY (Figure S3) between the HC, PSYCH, and NPFAM groups were calculated using the Kruskal–Wallis test (a nonparametric method for testing whether samples originate from the same distribution, which was used due to unequal variances in BACS between groups) after regressing out the effects of age, sex, data collection site, and the first ten principal components from the genetic ancestry analysis. These group differences were calculated between HC/PSYCH and HC/NPFAM instead of NPSYCH/PSYCH so that only unrelated individuals were compared. This was not a concern for correlation analyses within the NPSYCH group since the HC and the NPFAM subgroups were unrelated. Group differences in PRS_SCZ_ and PRS_EDUC_ were calculated between the HC and PSYCH groups (Fig. 1, Table S1, Kruskal–Wallis test was used due to unequal variance between groups for PRS_EDUC_) after regressing out the effects of data collection site and the first ten ancestry principal components. To correct for multiple hypotheses testing in analyses of the B-SNIP cohort a false discovery rate (FDR) approach^53^ was used following the example of recent studies that used polygenic risk scores^25,54^. For analyses with polygenic scores in B-SNIP the combined P_FDR-PRS_ was 0.0064 at *α* = 0.05. Analysis specific FDR *p*-values are reported with each result.

All analyses in the PNC were done using RStudio^55^ (Version 1.0.44). Since individuals in the PNC sample were controls and unrelated, correlations between polygenic scores and WRAT were calculated within the entire sample controlling for effects of age, sex, and the first 10 ancestry principal components using the Spearman Rank method. The FDR-corrected^53^ *p*-value threshold for PNC was P_FDR-PNC_ = 3.1 × 10^−12^ at α = 0.05 for all analyses using PRS_SCZ_ and PRS_EDUC_.

## Results

### Genetic risk for schizophrenia was higher among individuals with psychosis in the mixed diagnostic group in B-SNIP

An individual’s polygenic risk of schizophrenia, PRS_SCZ_, estimates genome-wide common genetic influences on the risk of developing schizophrenia. Compared to the HC (Fig. 1), individuals with psychosis from 3 diagnosis groups in the B-SNIP sample (schizophrenia, psychotic bipolar, schizoaffective disorder) showed significantly higher PRS_SCZ_ (*p* ≤ P_FDR_ _=_ 2.6 × 10^−4^, Table S1) at all P_T_. Among the psychosis probands schizophrenia patients had highest PRS_SCZ_ (Figure S4). In our sample PRS_EDUC_ did not differ significantly between the PSYCH and the HC groups (Table S1). Figure S4 shows the distributions of PRS_SCZ_ and PRS_EDUC_ for the different DSM diagnosis groups.

### Psychosis did not alter the correlations between EY, BACS, and WRAT in B-SNIP

**Fig. 2:**
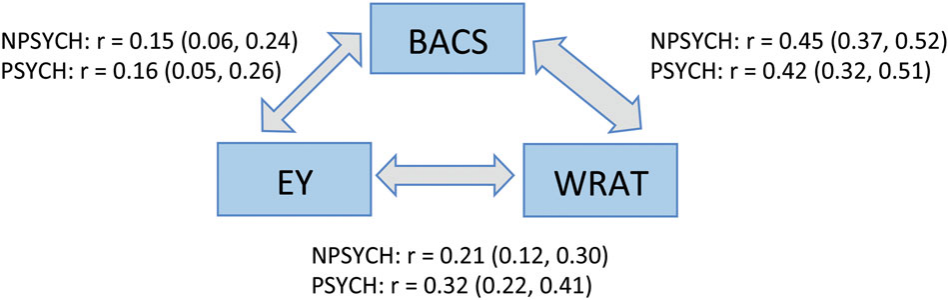
Relationship between the Brief Assessment of Cognition in Schizophrenia score (BACS), educational attainment (EY) and premorbid intellectual potential (WRAT) in B-SNIP. Correlation coefficients (Spearman’s Rank method) and 95% confidence intervals are shown. The three phenotypes were positively correlated in both the PSYCH (*N* = 314) and the NPSYCH (*N* = 423) groups and the magnitudes of the correlations were not significantly different between the groups. More details can be found in Table S2

An individual’s educational attainment, cognitive functioning and intellectual potential are interdependent traits^56^. We examined these relationships within the PSYCH and the NPSYCH groups separately in the B-SNIP sample and found that the presence of psychosis did not alter the extent to which the phenotypes are independent (Fig. 2). Although BACS, WRAT, and EY were significantly lower in the PSYCH group compared to the HC group (Figure S3), the effect size of deficit in BACS (Cohen’s *d* = 1.24, *p* = 8.1 × 10^−32^) was more than three times greater than that of EY or WRAT. Additionally, partial correlation analyses between pairs of these three phenotypes controlling for the third phenotype revealed that, (1) EY and WRAT shared a positive correlation that could not be accounted for by BACS; (2) WRAT and BACS shared a positive correlation that could not be accounted for by EY; and (3) although EY and BACS were weakly positively correlated, this correlation was mediated via factors that could be captured by WRAT (Table S2).

### Higher polygenetic risk for schizophrenia was significantly associated with lower BACS scores, but not WRAT or EY in nonpsychotic individuals in B-SNIP

**Fig. 3:**
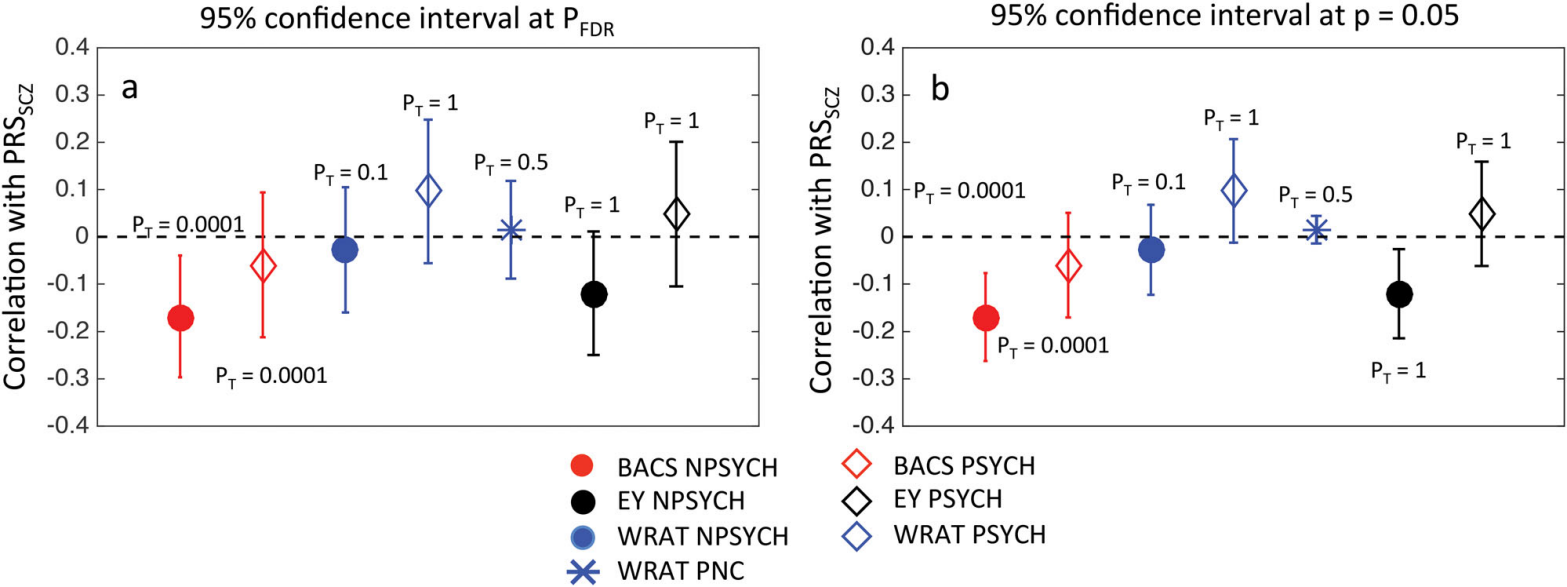
Correlations of the polygenic risk of schizophrenia (PRS_SCZ_) with the Brief Assessment of Cognition in Schizophrenia score (BACS), premorbid intellectual potential (WRAT) and educational attainment (EY). For B-SNIP: *N* = 314 (PSYCH), *N* = 423 (NPSYCH) and for the PNC: *N* = 4511. All markers other than the blue star, which represents PNC, show results for B-SNIP. Correlation coefficients are shown with 95% confidence intervals for FDR-corrected *p*-value threshold (*P*_FDR-B-SNIP_ = 0.0064, *P*_FDR-PNC_ = 3.1 × 10^−^^12^) (**a**) and *p* = 0.05 (**b**). Only the strongest correlation (Spearman’s Rank method) for each phenotype is shown with the corresponding P_T_ labeled. Correlation coefficients and corresponding *p*-values for all P_T_ can be found in Tables S3 and S6

To evaluate whether the genetic risk for schizophrenia associates with variations in BACS, EY, and WRAT, correlations of PRS_SCZ_ with these measures were calculated within the PSYCH and the NPSYCH groups in the B-SNIP sample separately. Figure 3 shows the strongest correlations for each phenotype. The numerical values for the correlation coefficients and the *p*-values for both groups at all P_T_ can be found in Table S3. BACS showed significant negative association with PRS_SCZ_ in the NPSYCH group (Fig. 3a, *r* = −0.17 and *p* = 6.6 × 10^−4^ at *P*_T_ = 1 × 10^−4^), but not in the PSYCH group. This association remained significant when variability due to EY and WRAT were accounted for by additionally controlling for those two phenotypes (Table S3). Nominally significant (*p* < 0.05) negative association was seen between PRS_SCZ_ and EY in the NPSYCH group, but not in the PSYCH group (Fig. 3b). WRAT was not significantly or nominally associated with PRS_SCZ_ in either group.

The correlation between BACS and PRS_SCZ_ within the schizophrenia proband group only (SZP, *N* = 100) was also not significant, similar to the results of the entire PSYCH group. Adding illness duration, number of hospitalization, chlorpromazine dose equivalent, number of psychotropic drugs, and social-functional scale score as covariates in the correlation analysis between BACS and PRS_SCZ_ did not alter the lack of significant results in the PSYCH group.

Since the NPSYCH group consisted of nonpsychotic individuals recruited as HC as well as the nonpsychotic family members of the psychosis probands (NPFAM, all subjects within this group were unrelated), the significance of the association of PRS_SCZ_ with BACS was additionally investigated within the HC and the NPFAM groups individually (Supplementary Material). At the subgroup level, statistically significant correlation (Table S4) between BACS and PRS_SCZ_ was seen at *P*_T_ = 10^−4^ in the HC group (*r* = −0.25, *p* = 1.9 × 10^−3^), which remained significant when EY and WRAT were regressed out. In the NPFAM subgroup, significant negative correlation was detected at P_T_ = 0.01 (*r* = −0.19, *p* = 6.4 × 10^−3^) when EY and WRAT were regressed out (Table S4).

**Fig. 4:**
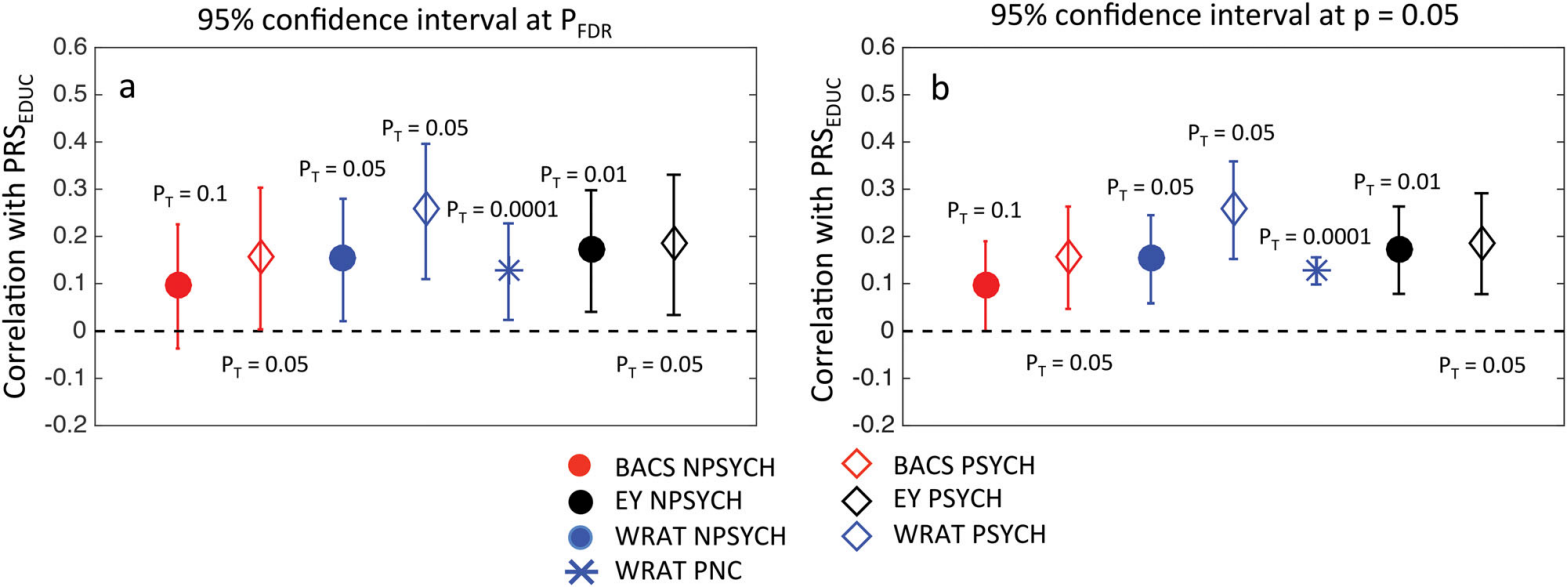
Correlations of the polygenic score of educational attainment (PRS_EDUC_) with the Brief Assessment of Cognition in Schizophrenia score (BACS), premorbid intellectual potential (WRAT), and educational attainment (EY). For B-SNIP: *N* = 314 (PSYCH), *N* = 423 (NPSYCH) and for PNC: *N* = 4511. All markers other than the blue star, which represents PNC, show results for B-SNIP. Correlation coefficients are shown with 95% confidence intervals for FDR-corrected threshold (*P*_FDR-B-SNIP_ = 0.0064, *P*_FDR-PNC_ = 3.1 × 10^−12^) (**a**) and *p* = 0.05 (**b**). Only the strongest correlation (Spearman’s Rank method) for each phenotype is shown with the corresponding P_T_ labeled. Correlation coefficients and corresponding *p*-values for all P_T_ can be found in Tables S5 and S6

The polygenic score of educational attainment, PRS_EDUC_, showed significant positive correlations with EY in both the PSYCH group (Fig. 4, strongest correlation of *r* = 0.19, *p* = 0.0016 at P_T_ = 0.05) and the NPSYCH group (Fig. 4, strongest correlation of *r* = 0.17, *p* = 7 × 10^−4^ at *P*_T_ = 0.01). Significant positive correlations were observed between PRS_EDUC_ and WRAT also in both the PSYCH group and the NPSYCH group (Fig. 4) at several P_T_ (strongest correlation of *r* = 0.26, *p* = 1.1 × 10^−5^ at *P*_T_ = 0.05 in PSYCH and strongest correlation of *r* = 0.15, *p* = 2.4 × 10^−3^ at *P*_T_ = 0.05 in NPSYCH). No significant correlation was found between PRS_EDUC_ and BACS in either group. The numerical values for all the correlation coefficients and *p*-values can be found in Table S5.

### Polygenic risk for schizophrenia and WRAT were unrelated in the PNC

We investigated the relationship between PRS_SCZ_, PRS_EDUC_, and WRAT in unrelated healthy individuals from the Philadelphia Neurocognitive Cohort (*N* = 4511). Our analyses showed lack of significant association between PRS_SCZ_ and WRAT at all P_T_ (Table S6, Fig. 3, *p* > 0.3 at all P_T_) and significant positive association between PRS_EDUC_ and WRAT at all P_T_ (Fig. 4, Table S6, *p* ≤ *P*_FDR-PNC_ = 3.1 × 10^−12^ at all P_T_; maximum correlation of *r* = 0.13 and *p* < 2.2 × 10^−16^ at *P*_T_ = 1 × 10^−4^). These results were consistent with the findings in the B-SNIP cohort.

## Discussion

Cognitive deficits are widespread in psychotic disorder patients, especially in schizophrenia. Recent molecular genetics studies have shown that schizophrenia is genetically negatively correlated with multiple measures of cognition^25,57^. Our work focused on the relationship between the common polygenic risk of schizophrenia and three cognitive measures that are phenotypically correlated (Fig. 2), but differentially associated (Fig S3) with psychosis-spectrum case status. Our main findings were: (1) BACS, a measure of general cognitive function (most strongly affected in the patient group) was negatively associated with the polygenic risk of schizophrenia in apparently healthy individuals, (2) WRAT, often used as a measure of premorbid intelligence in psychosis-spectrum patients, was not associated with the common genetic risk of schizophrenia in healthy or psychotic individuals, and (3) the negative association between BACS and the polygenic risk of schizophrenia did not appear to hold in the psychotic patient group.

The first finding is consistent with other recent reports of genetic overlap between general cognitive function and schizophrenia^24,25,57^. For example, Trampush et al.^57^, reported a genetic correlation of −0.17 between schizophrenia and general intelligence. Our B-SNIP sample was not large enough for applying the recently developed methods of LD Score Regression^58^ or GCTA^49^ to calculate genetic correlation between traits. However, PRS correlations to phenotype are mathematically translatable to genetic correlations between two traits^59^. In other words, a positive PRS association would translate to a positive genetic correlation of estimable magnitude. Hence, the negative association between PRS_SCZ_ and BACS is consistent with the above-mentioned negative genetic correlations between cognitive measures and schizophrenia^25,37^. Such negative associations have been shown in young cohorts also—Riglin et al.^60^ recently showed that lower performance intelligence quotient is associated with higher common genetic risk of schizophrenia in 14,701 samples of the ALSPAC cohort (age 7–9 years). Also, within the PNC cohort, it has been shown previously that the common genetic risk of schizophrenia negatively influences speed of verbal reasoning and emotion identification^39^.

The second and third findings were intriguing and warrant further investigation. In spite of sharing a phenotypic correlation of 0.4 with BACS, WRAT did not associate even nominally with the genetic risk of schizophrenia in either of our cohorts, including the large PNC. Although both BACS and WRAT measure cognitive function, BACS measures an individual’s ability to use cognitive resources to solve problems and WRAT is more of a measure of crystallized verbal knowledge. These results indicate the possibility that cognitive domains measured by BACS—rather than other brain phenotypes that shape premorbid intelligence—may be more direct targets of the genetic risk factors of schizophrenia.

Though we observed a significant negative correlation between PRS_SCZ_ and BACS at multiple P_T_ in the nonpsychotic group, we did not observe such a correlation among psychosis patients. Cognitive deficits in the patient group may thus reflect morbid factors such as, disease progression, protective effects of supportive care, and the effects of medications, medical, and psychiatric comorbidity and substance use, that are not predicted by PRS_SCZ_. However, recently, a similar result was reported in a study of Autism Spectrum Disorder (ASD) in which the polygenic risk of ASD did not predict IQ in the ASD probands (despite a strong positive correlation in the general population^61^), although the polygenic scores of educational attainment and schizophrenia did^62^. This too could be due to factors other than the genetic risk of developing the disease playing a significant role in determining the pathologic trajectory of cognitive function in the ASD patients.

Due to the relatively small sample size of the B-SNIP cohort, one might be concerned about statistical power of these analyses. In the B-SNIP cohort 80% statistical power corresponded to a correlation of magnitude 0.2 in the PSYCH group (*N* = 314) and of 0.17 in the NPSYCH group at *P*_FDR_ = 0.0064. The PRS_SCZ_ used here now explains ~20% or more of case-control variation in schizophrenia risk^19^, but it is difficult to estimate the expected relationship between that PRS and the specific domains of cognition considered here. It is particularly difficult because domains of cognition likely differ in their relationships with schizophrenia^25,39^. The genetic associations to the BACS should be replicated in a larger sample.

The polygenic score for educational attainment (PRS_EDUC_) showed significant positive association with years of education in both the psychotic and the nonpsychotic groups in B-SNIP, and also showed significant association with WRAT in both B-SNIP and PNC. However, the association between PRS_EDUC_ and BACS was not significant in the nonpsychotic group and was nominally significant (*p* < 0.05, Table S5) in the psychotic group in B-SNIP. While this lack of significant association with BACS in our work could be due to the relatively small B-SNIP sample size (BACS was not available in PNC), it is also possible that different cognitive domains have varying degrees of genetic overlap with educational attainment, and that cognitive phenotypes that assess verbal abilities are more closely genetically linked to educational performance. For example, in the UK Biobank data Hagenaars et al.^25^ reported strong genetic correlation between educational attainment and verbal-numerical reasoning (*r*_g_ ~ 0.72), but the genetic correlation of educational attainment with memory and reaction time were not significant.

Our results indicate the need to further explore the relationship between cognitive performance and the genetic risk factors of psychiatric disorders in larger patient groups. These findings also suggest that specific domains of cognition may be more closely etiologically linked to schizophrenia than other domains are, creating an opportunity for longitudinal studies to identify the domains that best predict illness onset.

## Electronic supplementary material

Supplementary Material
