# Supplementary material for: Polygenic risk for schizophrenia and measured domains of cognition in individuals with psychosis and controls
Source: Transl Psychiatry. 2018 Apr 12;8:78. doi: 10.1038/s41398-018-0124-8 (PMC5895806; doi:10.1038/s41398-018-0124-8)
Supplement: Supplementary file 1 — Supplementary Material [file 41398_2018_124_MOESM1_ESM.doc]

**Supplementary Material**

**Pre-imputation quality control of the B-SNIP and the PNC genotype data**

The B-SNIP genotypes underwent quality control using PLINK 1.9^40,41^ based on a standardized protocol^42^ in which individual markers were removed if they had a missing rate greater than 5%, deviated from Hardy-Weinberg equilibrium (p < 10^-6^), had a very low minor allele frequency (MAF < 0.01), or demonstrated significantly different call rates between psychiatric probands and controls (p < 10^-5^). Subjects were removed for discordant sex information, outlying heterozygosity (> 3 standard deviations above the mean), or excessive missing genotype data (> 0.1). Kinship analyses were run using both PREST-plus^63^ and KING^64^ to check for cryptic relatedness between unrelated individuals. Samples showing a relationship closer than 3rd degree to another unrelated individual, samples that failed or showed a misrepresented kinship and monozygotic twins were excluded resulting in 1962 remaining samples. Only samples with nonmissing age, sex, data collection site, BACS, WRAT, education, and history of psychosis were retained resulting in 1528 samples remained of whom 927 were Self Reported Caucasians (SRC). The ancestries of these SRC samples were verified by principal component analysis (Figure S1) combining the B-SNIP genotype data with the 1000 Genomes phase 1 data^43^. Samples that were more than four standard deviations away from the SRC group mean along the first ten principal components were excluded resulting in a final sample size of 737.

Genotype data for PNC were downloaded from dbGAP and quality controlled in PlLINK^40,41^ and GCTA^49^. Genotype data was distributed across five different Illumina genotyping chips (Human610_Quadv1 (2), HumanHap550v1 (2), HumanHap550v3 (2), HumanOmniExpress-12v1 (3), Human1M-Duov3_B (1); number of dbGAP datasets with each chip specified in parentheses). For each of the 10 datasets, quality control was performed in the following order: 1) remove markers with genotyping rate < 90% and minor allele frequency less than 0.01%, 2) remove samples missing more than 10% of variant calls, 3) exclude samples failing sex check, 4) keep markers with genotyping rate > 95% per marker, 5) exclude markers out of Hardy-Weinberg Equilibrium (p < 10^-10^), 6) Keep autosomal variants and remove duplicates, 7) Keep variants with minor allele frequency greater than 1%. All chips were merged together after per chip QC (tri-allelic variants removed from merged dataset) and the following QC was performed on the merged data: 1) retain variants with genotyping rate > 95% and minor allele frequency greater than 1%, 2) remove samples missing more than 10% of variant calls, 3) exclude samples failing sex check, 4) exclude samples with relatedness greater than pi-hat = 0.2, 5) retain variants with genotyping rate > 99%, 6) exclude variants out of Hardy-Weinberg Equilibrium (p < 10^-6^), 7) exclude samples with excess heterozygosity (< -0.05 or > 0.1), 8) remove markers with minor allele frequency less than 5%, 9) exclude non-single nucleotide polymorphism (SNP) variants, 10) retain samples of European ancestry by visual inspection against Hapmap reference (CEU, TSI, see Figure S2), 11) remove duplicate markers and markers with alleles failing to match reference genomes. After these filters were applied, 4,733 samples and 204,597 markers were retained for imputation.

**Group differences in EY, WRAT and BACS in B-SNIP**

Detailed differences in BACS performance between various proband groups and their family members and healthy controls were reported in^27^. In this work, we used a subset of these original samples, as our analysis was limited to the Caucasian samples only. Group differences in EY, WRAT and BACS for the sample used in this work are shown in Figure S3. For this analysis, P_FDR_ = 0.022. The NPFAM (nonpsychotic family members of PSYCH group members) and HC members of the NPSYCH group were treated separately in this case-control group comparison analysis so that only unrelated individuals were compared. Compared to the HC group, the PSYCH group displayed significantly lower EY, BACS and WRAT scores. The difference was most prominent for BACS (Cohen’s d = 1.24, p = 8.1 × 10^-32^) and remained highly significant (Cohen’s d = 0.97, p = 1.0 × 10^-32^) when EY and WRAT were adjusted for. The nonpsychotic family member group (NPFAM) showed lower BACS and EY compared to the HC group, although the effect sizes were smaller compared to those for the comparison between the HC and the PSYCH groups.

**Results within the HC and the NPFAM subgroups in B-SNIP**

Due to the relatively small sample size, nonpsychotic family members (NPFAM, N = 243) and the healthy controls (HC, N = 180) in B-SNIP were combined to form the NPSYCH group (N = 423). To account for spurious correlations that might arise from merging the two subgroups which differ in BACS (P_FDR_ = 0.022; higher BACS in HC, Cohen’s d = 0.27, group difference p = 0.0075, Figure S3) and PRS_SCZ_ (lower PRS_SCZ_ in HC, Cohen’s |d| <0.25, group difference p > 0.01 for all P_T_), the subjects’ HC or NPFAM status was used as a covariate for all within NPSYCH group correlation analyses. When the relationship between PRS_SCZ_ and BACS was explored within the HC and the NPFAM subgroups separately, there was no significant difference in the correlation coefficients of the two subgroups (Table S4). This indicates that the significant negative correlation between PRS_SCZ_ and BACS in the NPSYCH group was not driven by only one of the subgroups and was unlikely to be an artifact of combining the two subgroups into one group. At the subgroup level, statistically significant correlations (p ≤ P_FDR-PRS_ = 0.0064) were seen at P_T_ = 10^-4^ in the HC group (r = -0.25, p = 1.9 × 10^-3^). This correlation remained significant when EY and WRAT were regressed out (r = -0.27, p = 6.0 × 10^-4^). In the NPFAM group significant negative correlation was detected at P_T_ = 0.01 (r = - 0.19, p = 6.4 × 10^-3^) when EY and WRAT were regressed out.

**Figure Legends**

**Figure S1**: Ancestry verification of self-reported Caucasians in the B-SNIP data. Principal component analysis was performed on the genotype data combining the B-SNIP sample with the 1000 Genome data^43^. This figure shows a scatter plot of the first two principal components. The B-SNIP self-reported Caucasians (SRC) used in this study are shown in red (N = 737). The different ethnic groups of the 1000 Genome data are shown in black.

**Figure S2**: Ancestry analysis in the PNC cohort. Principal Component analysis was performed on the PNC genotype data merged with the Hapmap data^50^. The PNC samples are shown in magenta. The vertical and horizontal red lines correspond to boundaries for inclusion within the European ancestry group (N = 4511, all PNC samples in the top right block created by the red lines).

**Figure S3**: Mean values of education (EY), premorbid intellectual potential (WRAT) and cognition (BACS) in the PSYCH (N = 314), HC (N = 180) and NPFAM (N= 243) groups in B-SNIP. The HC and the NPFAM (nonpsychotic family members of PSYCH group probands) members of the NPSYCH group were treated separately in this case-control group comparison analysis so that only unrelated individuals were compared. Values are reported after regressing out the effects of age, sex, data collection site, and first 10 ancestry principal components. Residuals of regression were converted to z-scores for ease of interpretation. The red horizontal lines, and the pink and purple shaded regions represent the sample means, 95% confidence intervals of the standard errors of the mean (SEM) and standard deviations, respectively. Individual jittered data points are shown using grey dots. Panels a, c and e show differences in BACS, WRAT and EY between the PSYCH and the HC groups, respectively. Panels b, d and f show differences in BACS, WRAT and EY between the HC and the NPFAM groups, respectively. P-values correspond to Kruskal-Wallis tests. Cohen’s d values (pooled variance) for group differences are also shown for each panel. Positive Cohen’s d values indicate a higher mean in the HC group. For this analysis, P_FDR_ = 0.022.

**Figure S4:** Polygenic scores of schizophrenia (PRS_SCZ_) and educational attainment (PRS_EDUC_) in the different DSM diagnosis groups in B-SNIP. BPP: bipolar proband (N = 143), BPR: bipolar relative (N = 106), HC: healthy control (N = 180), SADBPP: schizoaffective bipolar-type probands (N = 50), SADBPR: schizoaffective bipolar-type relative (N = 37), SADDEPP: schizoaffective depression-type proband (N = 21), SADDEPR: schizoaffective depression-type relative (N = 17), SZP: schizophrenia probands (N = 100), SZR: schizophrenia relative (N = 83). Scores were calculated at seven p-value thresholds (P_T_): 0.0001, 0.001, 0.01, 0.05, 0.1, 0.5, 1.0, which are shown in different colors in both panels. The vertical black lines correspond to the standard errors of the mean (SEM). All scores were z-transformed before mean and SEM calculation.

**Table S1**: **Difference in PRS_SCZ_ and PRS_EDUC_ between the PSYCH (N = 314) and the HC (N = 180) groups in the B-SNIP cohort.**

| P_T_ | PSYCH/HC  PRS_SCZ_ | | | PSYCH/HC  PRS_EDUC_ | | |
| --- | --- | --- | --- | --- | --- | --- |
|  | Cohen’s d | | p-value | Cohen’s d | p-value | |
| 1 × 10^-4^ | **0.35** | **2.6 × 10^-4^** | | -0.15 | | 0.27 |
| 1 × 10^-3^ | **0.43** | **1.4 × 10^-5^** | | -0.08 | | 0.55 |
| 0.01 | **0.49** | **6.1 × 10^-7^** | | -0.04 | | 0.42 |
| 0.05 | **0.47** | **1.7 × 10^-6^** | | -0.04 | | 0.65 |
| 0.1 | **0.46** | **3.5 × 10^-6^** | | -0.07 | | 0.44 |
| 0.5 | **0.44** | **8.7 × 10^-6^** | | -0.05 | | 0.49 |
| 1.0 | **0.44** | **1.1 × 10^-5^** | | -0.03 | | 0.64 |

*Note*: Entries shown in bold are significant at p ≤ P_FDR_ = 2.6 × 10^-4^ for this analysis. Group differences were calculated using a non-parametric ANOVA approach (Kruskal-Wallis method). Effects of age, sex, data collection site, and ancestry principal components were regressed out before difference calculation. P_T_: polygenic score calculation threshold. Positive Cohen’s d values correspond to higher value in the PSYCH group. NPFAM (nonpsychotic family members of PSYCH group probands) were excluded from this case-control group comparison analysis so that only unrelated individuals were compared.

**Table S2**: **Correlations between years of education (EY), cognition (BACS) and premorbid intellectual potential (WRAT) in the PSYCH (N = 314) and the NPSYCH (N = 423) groups in B-SNIP.**

| Correlation Between | Controlled Parameter | PSYCH | | NPSYCH | |
| --- | --- | --- | --- | --- | --- |
|  |  | Correlation  Coefficient  r | p-value | Correlation Coefficient  r | p-value |
| EY, WRAT |  | **0.32** | **5.2 × 10^-8^** | **0.21** | **2.8 × 10^-5^** |
| EY, WRAT | BACS | **0.28** | **2.4 × 10^-6^** | **0.16** | **1.1 × 10^-3^** |
| EY, BACS |  | **0.16** | **6.0 × 10^-3^** | **0.15** | **3.7 × 10^-3^** |
| EY, BACS | WRAT | 0.03 | 0.61 | 0.062 | 0.22 |
| WRAT, BACS |  | **0.42** | **1.0 × 10^-13^** | **0.45** | **3.5 × 10^-21^** |
| WRAT, BACS | EY | **0.40** | **1.4 × 10^-12^** | **0.44** | **9.8 × 10^-20^** |

*Note*: Entries shown in bold are significant at the FDR threshold p ≤ P_FDR_ = 6.0 × 10^-3^ for this analysis (Methods). The effects of age, sex, collection site, ancestry principal components, DSM diagnosis (for PSYCH), DSM diagnoses of respective probands (in NPFAM), and HC/NPFAM status (for NPSYCH) were regressed out for all analyses. Using these residuals full correlations as well as partial correlations controlling for the third phenotype (second column from left) were calculated.

**Table S3**: **Results of full and partial correlation analyses of PRS_SCZ_ with EY, WRAT and BACS in the PSYCH (N = 314) and the NPSYCH (N = 423) groups in B-SNIP.**

|  | Correlation between PRS_SCZ_ and BACS | | | | Correlation between PRS_SCZ_ and BACS controlling for (WRAT, EY) | | | |
| --- | --- | --- | --- | --- | --- | --- | --- | --- |
| P_T_ | PSYCH | | NPSYCH | | PSYCH | | NPSYCH | |
|  | r | p | r | p | r | p | r | p |
| 1 × 10^-4^ | -0.060 | 0.31 | **-0.17** | **6.6 ×10^-4^** | -0.081 | 0.17 | **-0.19** | **1.0×10^-4^** |
| 1 × 10^-3^ | 0.020 | 0.73 | **-0.15** | **3.1×10^-3^** | 2.1×10^-3^ | 0.97 | **-0.16** | **1.8×10^-3^** |
| 0.01 | 1.9 × 10^-3^ | 0.97 | **-0.14** | **4.4×10^-3^** | -0.017 | 0.77 | **-0.17** | **8.5×10^-4^** |
| 0.05 | 0.023 | 0.70 | -0.12 | 2.1×10^-2^ | -0.015 | 0.80 | -0.12 | 0.016 |
| 0.1 | -1.4 × 10^-3^ | 0.98 | -0.13 | 9.5×10^-3^ | -0.045 | 0.45 | -0.13 | 0.012 |
| 0.5 | 0.025 | 0.67 | -0.11 | 2.2×10^-2^ | -0.018 | 0.76 | -0.12 | 0.019 |
| 1.0 | 0.027 | 0.65 | -0.12 | 1.9×10^-2^ | -0.018 | 0.76 | -0.12 | 0.016 |
|  | Correlation between PRS_SCZ_ and EY | | | | Correlation between PRS_SCZ_ and EY  controlling for (WRAT, BACS) | | | |
| P_T_ | PSYCH | | NPSYCH | | PSYCH | | NPSYCH | |
|  | r | p | r | p | r | p | r | p |
| 1 × 10^-4^ | -0.012 | 0.84 | -0.085 | 9.5 × 10^-2^ | -0.020 | 0.73 | -0.080 | 0.11 |
| 1 × 10^-3^ | 0.044 | 0.46 | -0.060 | 0.23 | 0.033 | 0.58 | -0.051 | 0.32 |
| 0.01 | 0.012 | 0.84 | -0.046 | 0.36 | -5.9 × 10^-4^ | 0.99 | -0.042 | 0.40 |
| 0.05 | 0.032 | 0.58 | -0.084 | 9.6 × 10^-2^ | 6.9 × 10^-3^ | 0.91 | -0.078 | 0.13 |
| 0.1 | 0.022 | 0.71 | -0.11 | 2.4 × 10^-2^ | -5.5 × 10^-3^ | 0.93 | -0.10 | 0.041 |
| 0.5 | 0.046 | 0.44 | -0.11 | 2.2 × 10^-2^ | 0.017 | 0.78 | -0.11 | 0.029 |
| 1.0 | 0.049 | 0.41 | -0.12 | 1.6 × 10^-2^ | 0.019 | 0.74 | -0.11 | 0.022 |
|  | Correlation between PRS_SCZ_ and WRAT | | | | Correlation between PRS_SCZ_ and WRAT  controlling for (EY, BACS) | | | |
| P_T_ | PSYCH | | NPSYCH | | PSYCH | | NPSYCH | |
|  | r | p | r | p | r | p | r | p |
| 1 × 10^-4^ | 0.031 | 0.59 | 0.019 | 0.71 | 0.067 | 0.26 | 0.12 | 0.019 |
| 1 × 10^-3^ | 0.040 | 0.50 | -9.9 × 10^-3^ | 0.85 | 0.024 | 0.68 | 0.069 | 0.17 |
| 0.01 | 0.041 | 0.49 | 0.022 | 0.66 | 0.043 | 0.46 | 0.10 | 0.044 |
| 0.05 | 0.084 | 0.16 | -8.6 × 10^-3^ | 0.86 | 0.077 | 0.19 | 0.060 | 0.24 |
| 0.1 | 0.091 | 0.13 | -0.028 | 0.58 | 0.099 | 0.098 | 0.050 | 0.32 |
| 0.5 | 0.096 | 0.11 | -7.7 × 10^-3^ | 0.88 | 0.086 | 0.15 | 0.066 | 0.19 |
| 1.0 | 0.098 | 0.098 | -7.0 × 10^-3^ | 0.89 | 0.087 | 0.15 | 0.069 | 0.17 |

*Note*: Entries shown in bold are significant at p ≤ P_FDR-PRS_ = 6.4 × 10^-3^. r: correlation coefficient, p: p-value, P_T_: polygenic score calculation threshold. The effects of age, sex, collection site, ancestry principal components, DSM diagnosis (for PSYCH), DSM diagnoses of respective probands (in NPFAM), and HC/NPFAM status (for NPSYCH) were regressed out. Correlations were calculated using the Spearman-Rank method, which is sensitive to nonlinear monotonic relationships.

**Table S4: Correlation between PRS_SCZ_ and BACS in the healthy control (HC, N = 180) and the nonpsychotic family members (NPFAM, N = 243) subgroups of the NPSYCH group in B-SNIP**.

|  | Correlation between PRS_SCZ_ and BACS | | | | Correlation between PRS_SCZ_ and BACS controlling for (WRAT, EY) | | | |
| --- | --- | --- | --- | --- | --- | --- | --- | --- |
| P_T_ | HC | | NPFAM | | HC | | NPFAM | |
|  | r | p | r | p | r | p | r | p |
| 1 × 10^-4^ | **-0.25** | **1.9 × 10^-3^** | -0.16 | 0.02 | **-0.27** | **6.0 × 10^-4^** | -0.17 | 0.013 |
| 1 × 10^-3^ | -0.11 | 0.17 | -0.17 | 0.015 | -0.12 | 0.11 | -0.16 | 0.019 |
| 0.01 | -0.11 | 0.17 | -0.16 | 0.016 | -0.14 | 0.087 | **-0.19** | **6.4 × 10^-3^** |
| 0.05 | -0.11 | 0.18 | -0.12 | 0.072 | -0.10 | 0.26 | -0.13 | 0.050 |
| 0.1 | -0.09 | 0.26 | -0.17 | 0.013 | -0.069 | 0.39 | -0.17 | 0.0146 |
| 0.5 | -0.10 | 0.20 | -0.14 | 0.040 | -0.085 | 0.29 | -0.14 | 0.032 |
| 1.0 | -0.11 | 0.16 | -0.13 | 0.047 | -0.094 | 0.25 | -0.14 | 0.034 |

*Note*: Entries show in bold are significant at p ≤ P_FDR-PRS_ = 6.4 × 10^-3^. r: correlation coefficient, p: p-value, P_T_: polygenic score calculation threshold. The effects of age, sex, collection site, ancestry principal components, and probands’ DSM diagnoses (for NPFAM only) were regressed out. Correlations were calculated using the Spearman-Rank method, which is sensitive to nonlinear monotonic relationships.

**Table S5**: **Results for full and partial correlation analyses of PRS_EDUC_ with EY, WRAT and BACS in the PSYCH (N = 314) and the NPSYCH (N = 423) groups in B-SNIP.**

|  | Correlation between PRS_EDUC_ and BACS | | | | Correlation between PRS_EDUC_ and BACS controlling for (WRAT, EY) | | | | |
| --- | --- | --- | --- | --- | --- | --- | --- | --- | --- |
| P_T_ | PSYCH | | NPSYCH | | PSYCH | | | NPSYCH | |
|  | r | p | r | p | r | p | | r | p |
| 1 × 10^-4^ | 0.032 | 0.59 | 0.014 | 0.78 | -0.047 | 0.43 | | -0.029 | 0.56 |
| 1 × 10^-3^ | 0.10 | 0.077 | 0.048 | 0.34 | 0.013 | 0.82 | | -8.7 × 10^-3^ | 0.86 |
| 0.01 | 0.11 | 0.052 | 0.083 | 0.099 | 0.017 | 0.77 | | 0.024 | 0.64 |
| 0.05 | 0.16 | 8.1 × 10^-3^ | 0.089 | 0.075 | 0.049 | 0.41 | | 0.018 | 0.72 |
| 0.1 | 0.14 | 0.015 | 0.096 | 0.057 | 0.055 | 0.35 | | 0.037 | 0.47 |
| 0.5 | 0.14 | 0.016 | 0.066 | 0.19 | 0.069 | 0.25 | | -5.5 × 10^-3^ | 0.91 |
| 1.0 | 0.13 | 0.023 | 0.069 | 0.17 | 0.060 | 0.31 | | 3.5 × 10^-3^ | 0.94 |
|  | Correlation between PRS_EDUC_ and EY | | | | Correlation between PRS_EDUC_ and EY controlling for (WRAT, BACS) | | | | |
| P_T_ | PSYCH | | NPSYCH | | PSYCH | | NPSYCH | | |
|  | r | p | r | p | r | p | r | | p |
| 1 × 10^-4^ | 0.089 | 0.14 | 0.087 | 0.089 | 0.039 | 0.51 | 0.073 | | 0.15 |
| 1 × 10^-3^ | 0.13 | 0.030 | **0.14** | **5.3 × 10^-3^** | 0.066 | 0.27 | 0.12 | | 0.015 |
| 0.01 | 0.14 | 0.015 | **0.17** | **7.1 × 10^-4^** | 0.077 | 0.19 | **0.15** | | **3.1 × 10^-3^** |
| 0.05 | **0.19** | **1.6 × 10^-3^** | **0.15** | **3.9 × 10^-3^** | 0.11 | 0.058 | 0.12 | | 0.020 |
| 0.1 | 0.16 | 7.6 × 10^-3^ | 0.12 | 0.018 | 0.095 | 0.11 | 0.093 | | 0.066 |
| 0.5 | 0.12 | 0.038 | 0.12 | 0.021 | 0.067 | 0.26 | 0.089 | | 0.077 |
| 1.0 | 0.12 | 0.043 | 0.11 | 0.037 | 0.065 | 0.27 | 0.079 | | 0.12 |
|  | Correlation between PRS_EDUC_ and WRAT | | | | Correlation between PRS_EDUC_ and WRAT controlling for (EY, BACS) | | | | |
| P_T_ | PSYCH | | NPSYCH | | PSYCH | | | NPSYCH | |
|  | r | p | r | p | r | p | | r | p |
| 1 × 10^-4^ | **0.17** | **4.3 × 10^-3^** | 0.081 | 0.11 | 0.16 | 9.2 × 10^-3^ | | 0.071 | 0.16 |
| 1 × 10^-3^ | **0.21** | **3.1 × 10^-4^** | 0.11 | 0.026 | **0.16** | **6.3 × 10^-3^** | | 0.081 | 0.11 |
| 0.01 | **0.23** | **1.1 × 10^-4^** | 0.12 | 0.015 | **0.17** | **4.0 × 10^-3^** | | 0.072 | 0.16 |
| 0.05 | **0.26** | **1.1 × 10^-5^** | **0.15** | **2.4 × 10^-3^** | **0.18** | **2.8 × 10^-3^** | | 0.11 | 0.034 |
| 0.1 | **0.22** | **2.3 × 10^-4^** | 0.13 | 8.6 × 10^-3^ | 0.14 | 0.017 | | 0.085 | 0.093 |
| 0.5 | **0.18** | **1.6 × 10^-3^** | **0.15** | **3.0 × 10^-3^** | 0.12 | 0.051 | | 0.12 | 0.019 |
| 1.0 | **0.18** | **1.9 × 10^-3^** | **0.14** | **5.0 × 10^-3^** | 0.12 | 0.048 | | 0.11 | 0.030 |

*Note*: Entries show in bold are significant at p ≤ P_FDR-PRS_ = 6.4 × 10^-3^. r: correlation coefficient, p: p-value, P_T_: polygenic score calculation threshold. The effects of age, sex, collection site, ancestry principal components, DSM diagnosis (for PSYCH), DSM diagnoses of respective probands (in NPFAM), and HC/NPFAM status (for NPSYCH) were regressed out. Correlations were calculated using the Spearman-Rank method that is sensitive to nonlinear monotonic relationships.

**Table S6**: **Correlations of WRAT with PRS_SCZ_ and PRS_EDUC_ in the PNC cohort (N = 4511).**

|  | Correlation with WRAT in PNC | | | |
| --- | --- | --- | --- | --- |
| P_T_ | PRS_SCZ_ | | PRS_EDUC_ | |
|  | r | p | r | p |
| 1 × 10^-4^ | 0.0049 | 0.74 | **0.13** | **< 2.2 × 10^-16^** |
| 1 × 10^-3^ | 0.015 | 0.31 | **0.12** | **1.2 × 10^-15^** |
| 0.01 | 0.0083 | 0.58 | **0.12** | **1.1 × 10^-15^** |
| 0.05 | 0.011 | 0.47 | **0.12** | **1.3 × 10^-15^** |
| 0.1 | 0.015 | 0.31 | **0.12** | **1.7 × 10^-15^** |
| 0.5 | 0.015 | 0.31 | **0.11** | **1.0 × 10^-12^** |
| 1.0 | 0.013 | 0.38 | **0.10** | **3.1 × 10^-12^** |

*Note*: Entries shown in bold are significant at p ≤ P_FDR-PNC_ = 3.1 × 10^-12^. r: correlation coefficient, p: p-value, P_T_: polygenic score calculation threshold. The effects of age, sex, and first 10 ancestry principal components were regressed out. Correlations were calculated using the Spearman-Rank method.

Figure S1


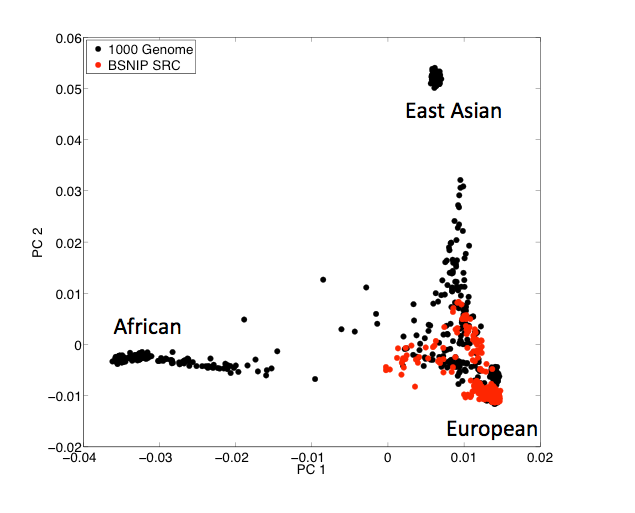


Figure S2


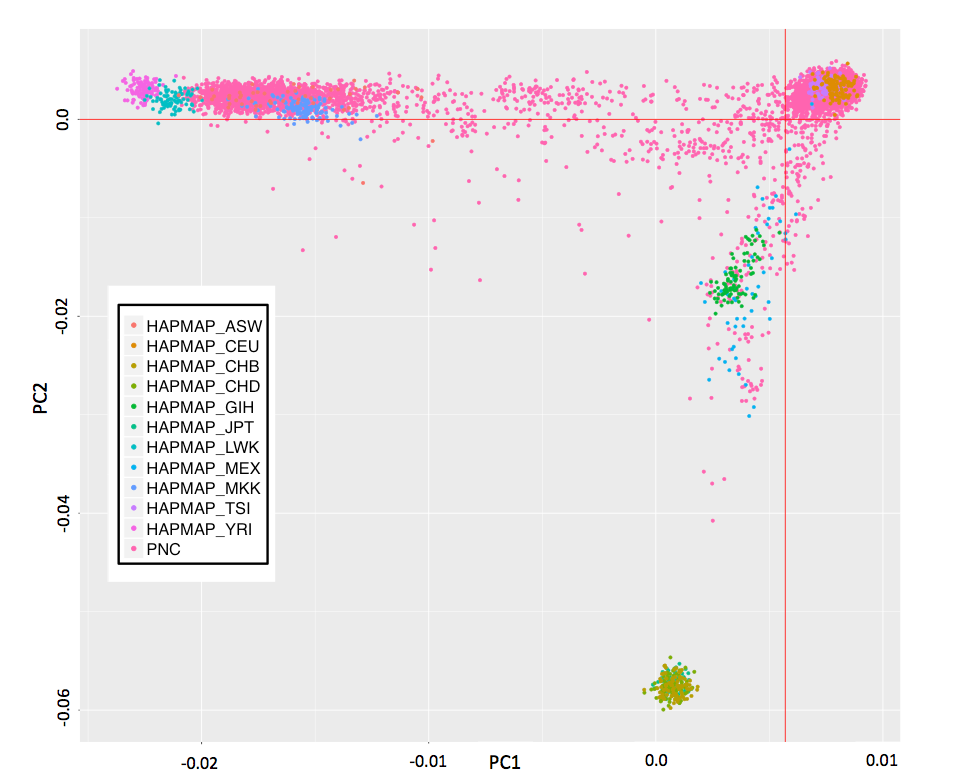


Figure S3


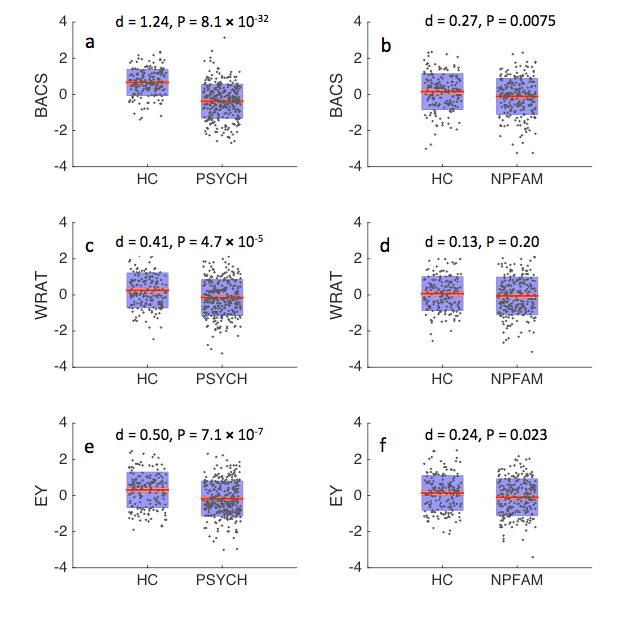


Figure S4


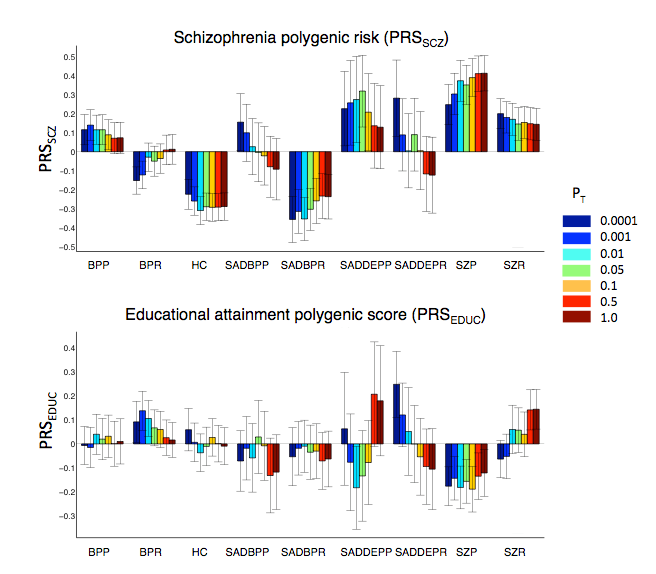


REFERENCES

1. Sun, L. & Dimitromanolakis, A. PREST-plus identifies pedigree errors and cryptic relatedness in the GAW18 sample using genome-wide SNP data. *BMC Proc*. **8**, S23 (2014). Suppl 1.
2. Manichaikul, A. et al. Robust relationship inference in genome-wide association studies. *Bioinformatics* **26**, 2867–2873 (2010).
